# 741 Occurrence and Outcomes of Burns in the Southeastern Diabetic Population

**DOI:** 10.1093/jbcr/irae036.284

**Published:** 2024-04-17

**Authors:** Sarah Moffitt, William West, Shreya Arora, Timothy Nehila, Bilal Koussayer, Nicole K Le, Kristen Whalen, Kristina Buller, Jared Troy, Jake Laun

**Affiliations:** Morsani College of Medicine USF, Tampa, FL; Morsani College of Medicine USF, Tampa, FL; Morsani College of Medicine USF, Tampa, FL; Morsani College of Medicine USF, Tampa, FL; Morsani College of Medicine USF, Tampa, FL; Morsani College of Medicine USF, Tampa, FL; Morsani College of Medicine USF, Tampa, FL; Morsani College of Medicine USF, Tampa, FL; Morsani College of Medicine USF, Tampa, FL; Morsani College of Medicine USF, Tampa, FL

## Abstract

**Introduction:**

Patients with diabetes typically account for 3 to 14% of burn patients. Diabetes augments the challenges presented to the body with a burn injury. Vascular injury, increased blood viscosity, and immune system dysfunction all put diabetic patients at a higher risk for developing an infection and poor wound healing. Also, the peripheral neuropathy associated with diabetes, dampening sensation, largely contributes to lower extremity burns during colder months when patients are using heating pads or warm compresses. There is conflicting evidence in the literature regarding predicted outcomes and hospital courses of diabetic burn patients. We analyzed how this prevalent comorbidity impacts health consequences among patients with burn injuries to strengthen treatment strategies and predict outcomes.

**Methods:**

A retrospective cohort analysis was performed on all patients between 2015-2020 over the age of 18 years that presented to our emergency department for burns. The data was obtained from the electronic medical record and statistical analysis was conducted using SPSS version 28.

**Results:**

Of the 1329 patients included, 14% were diabetic. 26% of diabetic patient burns experienced scald burns, while only 15% of nondiabetic burns were of that category (p< 0.01). Upper extremity burns were more common in non-diabetic patients (68%) compared to diabetic patients (50%) (p< 0.01). Nondiabetic patients presented statistically ≈1.5% lower area percentage of second-degree burns and ≈2.5% TBSA, compared to the diabetic population. We did not see any seasonal variations in burn incidents between the diabetic and non-diabetic populations. Having diabetes was not significantly correlated with having surgery, number of surgeries, surgical complications, infections, or being admitted to the ICU. Diabetes was a significant variable for increased length of stay (roughly 3 days longer when age, BMI, number of surgeries, total TBSA, and ICU days were held constant).

**Conclusions:**

The glycemic state of burn patients is important because of the influence of hyperglycemia on circulation, immune cell migration and function, and tissue repair. Our data recognize an extended hospital stay for diabetic patients, but without increased surgical complications. Moreover, we did not see a difference in burn occurrence for diabetic patients in colder months, likely due to the lack of cold temperatures in this region. Rather, we typically expect lower extremity burns in diabetics resulting from walking outside barefoot. Understanding diabetes as a comorbidity is crucial for preparing a treatment plan, particularly in burn injuries that strain the body’s healing capacity.

**Applicability of Research to Practice:**

Diabetes is a prevalent comorbidity that challenges the body's capability to heal. Understanding the impact diabetes has on burn outcomes can better prepare physicians to care for these patients.

